# Aqua­chlorido(4-methyl­benzoato-κ*O*)(1,10-phenanthroline-κ^2^ *N*,*N*′)copper(II)

**DOI:** 10.1107/S1600536808010945

**Published:** 2008-04-26

**Authors:** Wen-Dong Song, Hao Wang, Yan-Li Miao

**Affiliations:** a College of Science, Guang Dong Ocean University, Zhanjiang 524088, People’s Republic of China

## Abstract

In the title mononuclear complex, [Cu(C_8_H_7_O_2_)Cl(C_12_H_8_N_2_)(H_2_O)], the Cu^II^ atom is coordinated by one carboxylate O atom from a monodentate 4-methyl­benzoate ligand, two N atoms from the 1,10-phenanthroline ligand, one chloride ion and one water mol­ecule in a square-pyramidal geometry. The crystal structure exhibits inter- and intra­molecular C—H⋯Cl, C—H⋯O, O—H⋯Cl and O—H⋯O hydrogen bonds, as well as C—H⋯π inter­actions of phenanthroline and methyl H atoms towards the π-systems of neighboring 4-methyl­benzoate units and the pyridine rings of the phenanthroline system [centroid–centroid distances are 2.706 (2) and 2.992 (1) Å, respectively].

## Related literature

For related literature, see: Song *et al.* (2007 ▶).
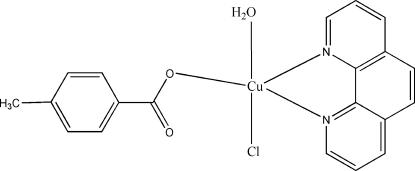


## Experimental

### 

#### Crystal data

[Cu(C_8_H_7_O_2_)Cl(C_12_H_8_N_2_)(H_2_O)]
*M* *_r_* = 432.35
Monoclinic, 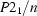

*a* = 10.9095 (4) Å
*b* = 11.0546 (4) Å
*c* = 15.2059 (6) Å
β = 103.578 (2)°
*V* = 1782.58 (12) Å^3^
*Z* = 4
Mo *K*α radiation
μ = 1.40 mm^−1^
*T* = 296 (2) K
0.30 × 0.29 × 0.25 mm

#### Data collection

Bruker APEXII area-detector diffractometer
Absorption correction: multi-scan (*SADABS*; Sheldrick, 1996 ▶) *T*_min_ = 0.679, *T*_max_ = 0.721
17233 measured reflections
4096 independent reflections
3470 reflections with *I* > 2σ(*I*)
*R*_int_ = 0.039

#### Refinement

*R*[*F* ^2^ > 2σ(*F* ^2^)] = 0.031
*wR*(*F* ^2^) = 0.087
*S* = 1.05
4096 reflections
251 parameters
3 restraints
H atoms treated by a mixture of independent and constrained refinement
Δρ_max_ = 0.36 e Å^−3^
Δρ_min_ = −0.41 e Å^−3^

### 

Data collection: *APEX2* (Bruker, 2004 ▶); cell refinement: *APEX2*; data reduction: *APEX2*; program(s) used to solve structure: *SHELXS97* (Sheldrick, 2008 ▶); program(s) used to refine structure: *SHELXL97* (Sheldrick, 2008 ▶); molecular graphics: *DIAMOND* (Brandenburg, 2001 ▶); software used to prepare material for publication: *SHELXTL*.

## Supplementary Material

Crystal structure: contains datablocks I, global. DOI: 10.1107/S1600536808010945/zl2109sup1.cif

Structure factors: contains datablocks I. DOI: 10.1107/S1600536808010945/zl2109Isup2.hkl

Additional supplementary materials:  crystallographic information; 3D view; checkCIF report

## Figures and Tables

**Table 1:** Hydrogen-bond geometry (Å, °)

*D*—H⋯*A*	*D*—H	H⋯*A*	*D*⋯*A*	*D*—H⋯*A*
C10—H10⋯Cl1^i^	0.93	2.76	3.654 (2)	162
C1—H1⋯O1	0.93	2.52	2.988 (3)	112
O1*W*—H2*W*⋯Cl1^i^	0.816 (10)	2.259 (10)	3.0709 (17)	174 (3)
O1*W*—H1*W*⋯O2	0.812 (10)	1.788 (14)	2.526 (2)	150 (3)
C3—H3⋯*Cg*1^ii^	0.93	2.71	3.413 (2)	133
C20—H20*B*⋯*Cg*2^iii^	0.93	2.99	3.627 (2)	125

Symmetry codes: (i) 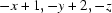; (ii) 
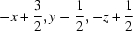
; (iii) 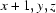. *Cg*1 and *Cg*2 are the centroids of the C14–C19 and C1–C4,C12,N1 rings, respectively.

**Fig. 1.**
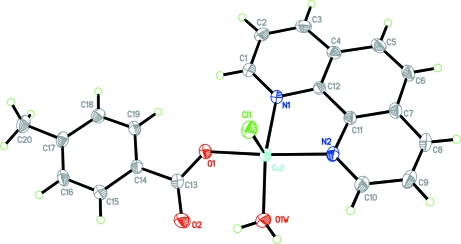
The structure of (I), showing the atomic numbering scheme. Non-H atoms are shown with 30% probability displacement ellipsoids.

**Fig. 2.**
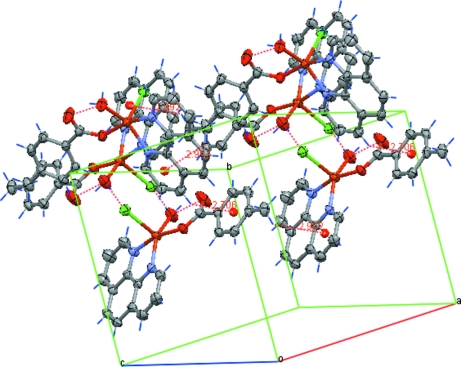
A packing view of the title compound. The intermolecluar hydrogen bonds and C—H···π interactions are shown as dashed lines.

## supplementary crystallographic information

### Comment

In the structural investigation of 4-methylbenzoate complexes, it has been found that the 4-methylbenzoic acid functions as a multidentate ligand [Song *et al.* (2007)], with versatile binding and coordination modes. In this paper, we report the crystal structure of the title compound, (I), a new Cu complex obtained by the reaction of 4-methylbenzoic acid, 1,10-phenanthroline and copper chloride in an alkaline aqueous solution.

As illustrated in Figure 1, the Cu^II^ atom exists in a square pyramidal environment, defined by one carboxyl O atom from a monodentate 4-methylbenzate ligand, two N atoms from the 1,10-phenanthroline ligand, one chlorine ion and a water molecule. The crystal structure exhibits inter and intramolecular C—H···Cl, C—H···O, O—H···Cl and O—H···O hydrogen bonding and C—H···π interactions of phenanthroline and methyl H atoms towards the π-systems of neighboring 4-methylbenzate units and of pyridine rings of the phenanthroline system. Centroid to centroid distances are 2.706 (2)Å and 2.992 (1) Å, respectively. (Table 1, Fig. 2, Cg1 = Ring (C14-C19) ; Cg2 = Ring (C1-C4 ,C12, N1)).

### Experimental

A mixture of copper chloride (1 mmol), 4-methylbenzoic acid (1 mmol), phen (1 mmol), NaOH (1.5 mmol) and H_2_O (12 ml) was placed in a 23 ml Teflon reactor, which was heated to 433 K for three days and then cooled to room temperature at a rate of 10 K h^-1^. The crystals obtained were washed with water and dryed in air.

### Refinement

H atoms were placed at calculated positions and were treated as riding on the parent C atoms with C—H = 0.93 - 0.97 Å, N—H = 0.86 Å, and with *U*_iso_(H) = 1.2 *U*_eq_(C, N).

### Crystal data

**Table:**

[Cu(C_8_H_7_O_2_)Cl(C_12_H_8_N_2_)(H_2_O)]	*F*_000_ = 884
*M**_r_* = 432.35	*D*_x_ = 1.611 Mg m^−^^3^
Monoclinic, *P*2_1_/*n*	Mo *K*α radiation λ = 0.71073 Å
Hall symbol: -P 2yn	Cell parameters from 2895 reflections
*a* = 10.9095 (4) Å	θ = 2.4–27.9º
*b* = 11.0546 (4) Å	µ = 1.40 mm^−^^1^
*c* = 15.2059 (6) Å	*T* = 296 (2) K
β = 103.578 (2)º	Block, blue
*V* = 1782.58 (12) Å^3^	0.30 × 0.29 × 0.25 mm
*Z* = 4	

### Data collection

**Table:**

Bruker APEXII area-detector diffractometer	4096 independent reflections
Radiation source: fine-focus sealed tube	3470 reflections with *I* > 2σ(*I*)
Monochromator: graphite	*R*_int_ = 0.039
*T* = 296(2) K	θ_max_ = 27.5º
φ and ω scans	θ_min_ = 2.1º
Absorption correction: multi-scan(SADABS; Sheldrick, 1996)	*h* = −13→14
*T*_min_ = 0.679, *T*_max_ = 0.721	*k* = −14→14
17233 measured reflections	*l* = −19→19

### Refinement

**Table:**

Refinement on *F*^2^	Secondary atom site location: difference Fourier map
Least-squares matrix: full	Hydrogen site location: inferred from neighbouring sites
*R*[*F*^2^ > 2σ(*F*^2^)] = 0.031	H atoms treated by a mixture of independent and constrained refinement
*wR*(*F*^2^) = 0.087	*w* = 1/[σ^2^(*F*_o_^2^) + (0.0424*P*)^2^ + 0.6484*P*] where *P* = (*F*_o_^2^ + 2*F*_c_^2^)/3
*S* = 1.05	(Δ/σ)_max_ = 0.001
4096 reflections	Δρ_max_ = 0.36 e Å^−^^3^
251 parameters	Δρ_min_ = −0.40 e Å^−^^3^
3 restraints	Extinction correction: none
Primary atom site location: structure-invariant direct methods	

### Special details

**Table:**

Geometry. All e.s.d.'s (except the e.s.d. in the dihedral angle between two l.s. planes) are estimated using the full covariance matrix. The cell e.s.d.'s are taken into account individually in the estimation of e.s.d.'s in distances, angles and torsion angles; correlations between e.s.d.'s in cell parameters are only used when they are defined by crystal symmetry. An approximate (isotropic) treatment of cell e.s.d.'s is used for estimating e.s.d.'s involving l.s. planes.
Refinement. Refinement of *F*^2^ against ALL reflections. The weighted *R*-factor *wR* and goodness of fit *S* are based on *F*^2^, conventional *R*-factors *R* are based on *F*, with *F* set to zero for negative *F*^2^. The threshold expression of *F*^2^ > σ(*F*^2^) is used only for calculating *R*-factors(gt) *etc*. and is not relevant to the choice of reflections for refinement. *R*-factors based on *F*^2^ are statistically about twice as large as those based on *F*, and *R*- factors based on ALL data will be even larger.

### Fractional atomic coordinates and isotropic or equivalent isotropic displacement parameters (Å^2^)

**Table:**

	*x*	*y*	*z*	*U*_iso_*/*U*_eq_	
C1	0.64907 (19)	0.5567 (2)	0.17000 (14)	0.0353 (4)	
H1	0.7299	0.5880	0.1905	0.042*	
C2	0.6213 (2)	0.4448 (2)	0.20277 (15)	0.0417 (5)	
H2	0.6826	0.4030	0.2445	0.050*	
C3	0.5034 (2)	0.3969 (2)	0.17311 (14)	0.0401 (5)	
H3	0.4843	0.3214	0.1934	0.048*	
C4	0.41115 (19)	0.46272 (19)	0.11182 (13)	0.0323 (4)	
C5	0.2842 (2)	0.4229 (2)	0.07897 (14)	0.0387 (5)	
H5	0.2592	0.3489	0.0980	0.046*	
C6	0.1996 (2)	0.4917 (2)	0.02029 (15)	0.0396 (5)	
H6	0.1174	0.4638	−0.0003	0.047*	
C7	0.23384 (19)	0.6059 (2)	−0.01065 (14)	0.0349 (4)	
C8	0.1518 (2)	0.6819 (2)	−0.07119 (17)	0.0470 (6)	
H8	0.0687	0.6587	−0.0949	0.056*	
C9	0.1943 (2)	0.7895 (2)	−0.09500 (19)	0.0541 (7)	
H9	0.1399	0.8406	−0.1345	0.065*	
C10	0.3190 (2)	0.8236 (2)	−0.06052 (16)	0.0440 (5)	
H10	0.3463	0.8979	−0.0775	0.053*	
C11	0.35799 (17)	0.64676 (18)	0.02085 (12)	0.0287 (4)	
C12	0.44711 (17)	0.57430 (17)	0.08258 (12)	0.0276 (4)	
C13	0.85765 (19)	0.81625 (19)	0.06922 (14)	0.0343 (4)	
C14	0.98797 (18)	0.77868 (18)	0.11736 (13)	0.0305 (4)	
C15	1.09054 (19)	0.8525 (2)	0.11423 (15)	0.0375 (5)	
H15	1.0786	0.9222	0.0790	0.045*	
C16	1.20987 (19)	0.8220 (2)	0.16345 (15)	0.0397 (5)	
H16	1.2773	0.8727	0.1614	0.048*	
C17	1.23162 (19)	0.7185 (2)	0.21550 (14)	0.0354 (5)	
C18	1.12943 (19)	0.6429 (2)	0.21623 (14)	0.0364 (5)	
H18	1.1422	0.5713	0.2492	0.044*	
C19	1.00983 (19)	0.67339 (19)	0.16855 (14)	0.0336 (4)	
H19	0.9426	0.6226	0.1707	0.040*	
C20	1.3607 (2)	0.6881 (3)	0.27276 (16)	0.0476 (6)	
H20A	1.4229	0.7369	0.2541	0.071*	
H20B	1.3784	0.6042	0.2654	0.071*	
H20C	1.3629	0.7040	0.3352	0.071*	
Cl1	0.55415 (5)	0.92640 (5)	0.16915 (4)	0.03802 (13)	
Cu1	0.58608 (2)	0.78128 (2)	0.052071 (16)	0.02995 (9)	
N1	0.56500 (14)	0.62052 (15)	0.11079 (10)	0.0290 (3)	
N2	0.40076 (16)	0.75369 (16)	−0.00414 (12)	0.0327 (4)	
O1	0.76822 (13)	0.76408 (14)	0.09531 (10)	0.0352 (3)	
O2	0.84387 (16)	0.8934 (2)	0.00865 (14)	0.0697 (6)	
O1W	0.60789 (15)	0.87990 (17)	−0.05137 (12)	0.0500 (4)	
H1W	0.6788 (12)	0.908 (2)	−0.041 (2)	0.075*	
H2W	0.563 (2)	0.9330 (19)	−0.0792 (19)	0.075*	

### Atomic displacement parameters (Å^2^)

**Table:**

	*U*^11^	*U*^22^	*U*^33^	*U*^12^	*U*^13^	*U*^23^
C1	0.0295 (10)	0.0428 (12)	0.0317 (10)	−0.0006 (9)	0.0034 (8)	0.0039 (9)
C2	0.0400 (12)	0.0482 (13)	0.0348 (11)	0.0060 (10)	0.0044 (9)	0.0126 (10)
C3	0.0479 (13)	0.0383 (12)	0.0354 (11)	−0.0028 (10)	0.0124 (9)	0.0073 (9)
C4	0.0370 (11)	0.0319 (10)	0.0296 (10)	−0.0045 (8)	0.0112 (8)	−0.0021 (8)
C5	0.0404 (12)	0.0388 (12)	0.0390 (11)	−0.0134 (9)	0.0132 (9)	−0.0029 (9)
C6	0.0306 (10)	0.0473 (13)	0.0404 (11)	−0.0141 (9)	0.0075 (9)	−0.0068 (10)
C7	0.0279 (10)	0.0428 (12)	0.0326 (10)	−0.0064 (8)	0.0042 (8)	−0.0054 (9)
C8	0.0257 (10)	0.0598 (15)	0.0492 (13)	−0.0063 (10)	−0.0041 (9)	0.0032 (12)
C9	0.0348 (12)	0.0607 (17)	0.0574 (16)	0.0031 (11)	−0.0078 (11)	0.0166 (13)
C10	0.0333 (11)	0.0433 (12)	0.0499 (13)	−0.0008 (10)	−0.0013 (10)	0.0133 (11)
C11	0.0263 (9)	0.0322 (10)	0.0271 (9)	−0.0029 (8)	0.0052 (7)	−0.0023 (8)
C12	0.0267 (9)	0.0329 (10)	0.0233 (8)	−0.0032 (8)	0.0059 (7)	−0.0025 (7)
C13	0.0283 (10)	0.0372 (11)	0.0355 (10)	−0.0047 (8)	0.0037 (8)	0.0011 (9)
C14	0.0263 (9)	0.0354 (11)	0.0295 (10)	−0.0033 (8)	0.0057 (8)	−0.0034 (8)
C15	0.0316 (10)	0.0395 (12)	0.0414 (11)	−0.0053 (9)	0.0083 (9)	0.0032 (9)
C16	0.0256 (10)	0.0486 (13)	0.0448 (12)	−0.0086 (9)	0.0080 (9)	−0.0058 (10)
C17	0.0260 (10)	0.0477 (13)	0.0317 (10)	0.0025 (9)	0.0054 (8)	−0.0089 (9)
C18	0.0342 (11)	0.0379 (11)	0.0362 (11)	0.0036 (9)	0.0067 (9)	0.0001 (9)
C19	0.0288 (10)	0.0360 (11)	0.0361 (10)	−0.0049 (8)	0.0081 (8)	−0.0038 (9)
C20	0.0290 (11)	0.0691 (16)	0.0419 (12)	0.0051 (11)	0.0026 (9)	−0.0065 (12)
Cl1	0.0337 (3)	0.0380 (3)	0.0397 (3)	0.0030 (2)	0.0033 (2)	0.0001 (2)
Cu1	0.02281 (13)	0.03156 (15)	0.03284 (15)	−0.00285 (9)	0.00123 (10)	0.00481 (10)
N1	0.0242 (8)	0.0334 (9)	0.0281 (8)	−0.0019 (7)	0.0035 (6)	0.0015 (7)
N2	0.0263 (8)	0.0349 (9)	0.0335 (9)	−0.0027 (7)	0.0002 (7)	0.0022 (7)
O1	0.0234 (7)	0.0412 (8)	0.0395 (8)	−0.0019 (6)	0.0048 (6)	0.0063 (6)
O2	0.0325 (9)	0.0915 (15)	0.0800 (13)	−0.0065 (9)	0.0027 (9)	0.0516 (12)
O1W	0.0322 (8)	0.0648 (11)	0.0498 (10)	0.0020 (8)	0.0033 (7)	0.0280 (9)

### Geometric parameters (Å, °)

**Table:**

C1—N1	1.327 (2)	C13—O2	1.238 (3)
C1—C2	1.394 (3)	C13—O1	1.274 (2)
C1—H1	0.9300	C13—C14	1.497 (3)
C2—C3	1.366 (3)	C14—C19	1.389 (3)
C2—H2	0.9300	C14—C15	1.395 (3)
C3—C4	1.403 (3)	C15—C16	1.382 (3)
C3—H3	0.9300	C15—H15	0.9300
C4—C12	1.398 (3)	C16—C17	1.381 (3)
C4—C5	1.427 (3)	C16—H16	0.9300
C5—C6	1.356 (3)	C17—C18	1.395 (3)
C5—H5	0.9300	C17—C20	1.509 (3)
C6—C7	1.428 (3)	C18—C19	1.377 (3)
C6—H6	0.9300	C18—H18	0.9300
C7—C11	1.401 (3)	C19—H19	0.9300
C7—C8	1.403 (3)	C20—H20A	0.9600
C8—C9	1.356 (3)	C20—H20B	0.9600
C8—H8	0.9300	C20—H20C	0.9600
C9—C10	1.390 (3)	Cl1—Cu1	2.4810 (6)
C9—H9	0.9300	Cu1—O1	1.9503 (14)
C10—N2	1.330 (3)	Cu1—O1W	1.9736 (16)
C10—H10	0.9300	Cu1—N2	2.0248 (17)
C11—N2	1.357 (3)	Cu1—N1	2.0261 (17)
C11—C12	1.428 (3)	O1W—H1W	0.812 (10)
C12—N1	1.357 (2)	O1W—H2W	0.816 (10)
			
N1—C1—C2	122.73 (19)	C16—C15—C14	120.0 (2)
N1—C1—H1	118.6	C16—C15—H15	120.0
C2—C1—H1	118.6	C14—C15—H15	120.0
C3—C2—C1	119.5 (2)	C17—C16—C15	121.7 (2)
C3—C2—H2	120.2	C17—C16—H16	119.1
C1—C2—H2	120.2	C15—C16—H16	119.1
C2—C3—C4	119.4 (2)	C16—C17—C18	118.09 (19)
C2—C3—H3	120.3	C16—C17—C20	121.8 (2)
C4—C3—H3	120.3	C18—C17—C20	120.1 (2)
C12—C4—C3	117.22 (18)	C19—C18—C17	120.6 (2)
C12—C4—C5	118.85 (19)	C19—C18—H18	119.7
C3—C4—C5	123.92 (19)	C17—C18—H18	119.7
C6—C5—C4	120.8 (2)	C18—C19—C14	121.09 (19)
C6—C5—H5	119.6	C18—C19—H19	119.5
C4—C5—H5	119.6	C14—C19—H19	119.5
C5—C6—C7	121.51 (19)	C17—C20—H20A	109.5
C5—C6—H6	119.2	C17—C20—H20B	109.5
C7—C6—H6	119.2	H20A—C20—H20B	109.5
C11—C7—C8	116.6 (2)	C17—C20—H20C	109.5
C11—C7—C6	118.62 (19)	H20A—C20—H20C	109.5
C8—C7—C6	124.78 (19)	H20B—C20—H20C	109.5
C9—C8—C7	119.7 (2)	O1—Cu1—O1W	91.07 (6)
C9—C8—H8	120.2	O1—Cu1—N2	164.79 (7)
C7—C8—H8	120.2	O1W—Cu1—N2	92.41 (7)
C8—C9—C10	120.2 (2)	O1—Cu1—N1	88.73 (6)
C8—C9—H9	119.9	O1W—Cu1—N1	152.03 (8)
C10—C9—H9	119.9	N2—Cu1—N1	81.29 (7)
N2—C10—C9	122.3 (2)	O1—Cu1—Cl1	97.16 (5)
N2—C10—H10	118.9	O1W—Cu1—Cl1	106.16 (6)
C9—C10—H10	118.9	N2—Cu1—Cl1	96.07 (5)
N2—C11—C7	123.57 (18)	N1—Cu1—Cl1	101.62 (5)
N2—C11—C12	116.53 (16)	C1—N1—C12	117.86 (17)
C7—C11—C12	119.89 (18)	C1—N1—Cu1	129.28 (14)
N1—C12—C4	123.21 (18)	C12—N1—Cu1	112.85 (12)
N1—C12—C11	116.44 (17)	C10—N2—C11	117.66 (18)
C4—C12—C11	120.35 (17)	C10—N2—Cu1	129.52 (15)
O2—C13—O1	125.12 (19)	C11—N2—Cu1	112.82 (13)
O2—C13—C14	119.36 (18)	C13—O1—Cu1	130.15 (14)
O1—C13—C14	115.52 (18)	Cu1—O1W—H1W	110 (2)
C19—C14—C15	118.41 (19)	Cu1—O1W—H2W	130 (2)
C19—C14—C13	121.65 (18)	H1W—O1W—H2W	105.0 (15)
C15—C14—C13	119.90 (19)		

### Hydrogen-bond geometry (Å, °)

**Table:**

*D*—H···*A*	*D*—H	H···*A*	*D*···*A*	*D*—H···*A*
C10—H10···Cl1^i^	0.93	2.76	3.654 (2)	162
C1—H1···O1	0.93	2.52	2.988 (3)	112
O1W—H2W···Cl1^i^	0.816 (10)	2.259 (10)	3.0709 (17)	174 (3)
O1W—H1W···O2	0.812 (10)	1.788 (14)	2.526 (2)	150 (3)
C3—H3···Cg1^ii^	0.93	2.71	3.413 (2)	133
C20—H20B···Cg2^iii^	0.93	2.99	3.627 (2)	125

Symmetry codes: (i) −*x*+1, −*y*+2, −*z*; (ii) −*x*+3/2, *y*−1/2, −*z*+1/2; (iii) *x*+1, *y*, *z*.
